# Supplementary material for: Spider-silk-inspired strong and tough hydrogel fibers with anti-freezing and water retention properties
Source: Nat Commun. 2024 May 24;15:4441. doi: 10.1038/s41467-024-48745-9 (PMC11126733; doi:10.1038/s41467-024-48745-9)
Supplement: Supplementary file 1 — Supplementary Information [file 41467_2024_48745_MOESM1_ESM.pdf]

Spider-silk-inspired strong and tough hydrogel fibers with anti-freezing and water retention properties

Shaoji Wu[1], Zhao Liu[1], Caihong Gong[1], Wanjiang Li[1], Sijia Xu[1], Rui Wen[1], Wen Feng[2, *], Zhiming Qiu[1], Yurong Yan[1, 3, *]

[1] School of Materials Science and Engineering, South China University of Technology, Guangzhou, 510641, P.R. China.

[2] Guangdong Medical Products Administration Key Laboratory for Quality Research and Evaluation of Medical Textile Products, Guangzhou, 511447, P.R. China.

[3] Key Lab of Guangdong High Property & Functional Polymer Materials, Guangzhou, 510640, P.R. China

* Corresponding authors E-mail: yryan@scut.edu.cn, fengw@gttc.net.cn

Table of contents

Supplementary Figure 1 --- 2

Supplementary Figure 2 --- 2

Supplementary Figure 3 --- 2

Supplementary Figure 4 --- 3

Supplementary Figure 5 --- 3

Supplementary Figure 6 --- 4

Supplementary Figure 7 --- 5

Supplementary Figure 8 --- 5

Supplementary Figure 9 --- 6

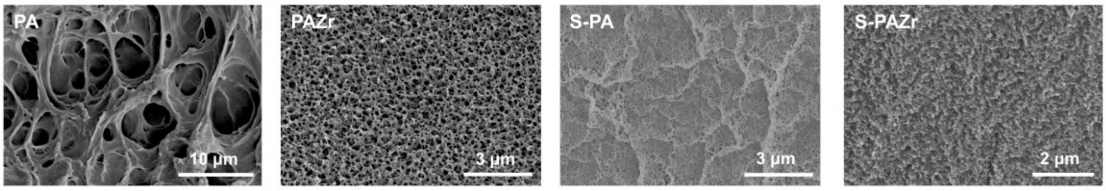

Supplementary Figure 1. SEM tests of PA, PAZr, S-PA, and S-PAZr. PVA/PAA (PA) without ionic crosslinking and non-salting-out treatment, S-PVA/PAA (S-PA) with salting-out treatment, PVA/PAA/Zr^{4+} (PAZr) with ionic crosslinking, and S-PVA/PAA/Zr^{4+} (S-PAZr) with ionic crosslinking and salting-out treatment.

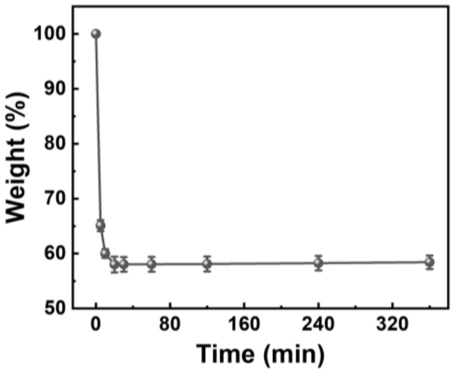

Supplementary Figure 2. Weight changes of PA hydrogel fibers converted to S-PAZr hydrogel fibers by immersion in a Na_2SO_4/Gly/H_2O ternary solvent. A large amount of water was lost during the conversion of the PAZr hydrogel fibers into S-PAZr hydrogel fibers. Data were presented as mean ± SD (n = 3 independent samples).

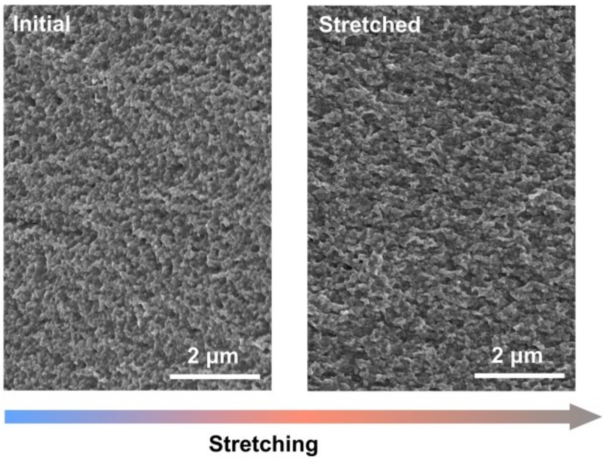

Supplementary Figure 3. SEM images of S-PAZr before and after stretching. The network structure of S-PAZr would be oriented during stretching.

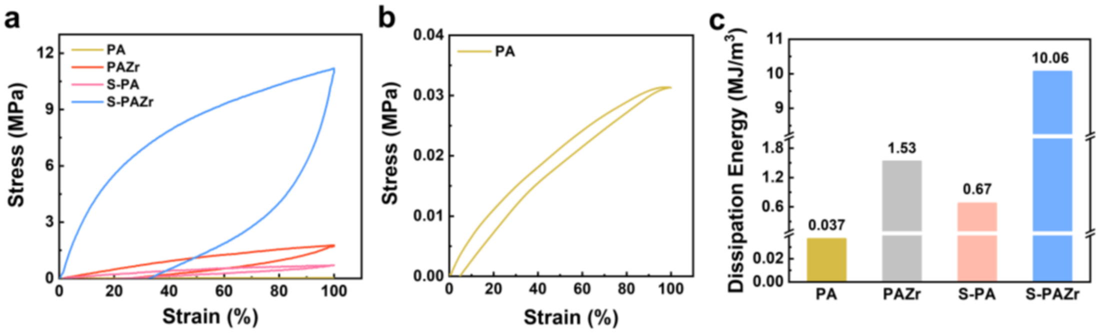

Supplementary Figure 4. (a-b) Loading-Unloading curves and (c) dissipation energy of PA, PAZr, S-PA, and S-PAZr hydrogel fibers. The dissipation energies of PAZr hydrogel fibers with ionic coordination and crystal domain crosslinked S-PA hydrogel fibers were 1.53 MJ/m^3 and 0.67 MJ/m^3, respectively, which were much higher than the 0.037 MJ/m^3 of the pristine PA hydrogel fibers. Remarkably, owing to the synergistic dissipation energies of ionic coordination and crystal domain crosslinking, the S-PAZr hydrogel exhibited a extremely high dissipation energy of 10.06 MJ/m^3.

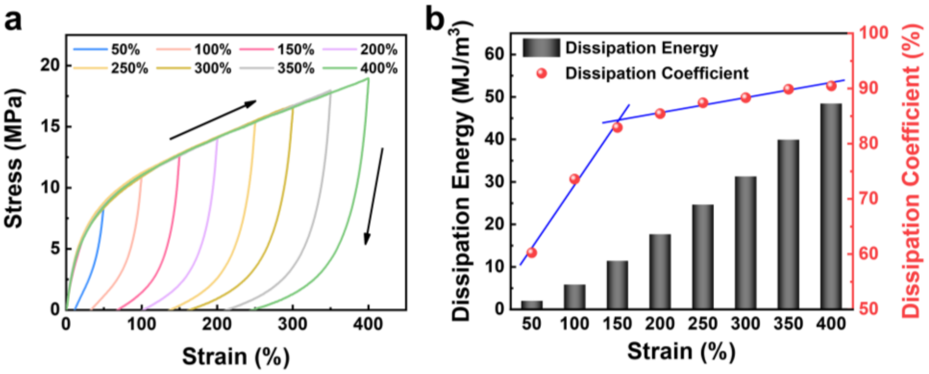

Supplementary Figure 5. (a) Loading-Unloading curves, (b) dissipation energy, and dissipation coefficent of S-PAZr hydrogel at different strains. With the increase of tensile strain, the S-PAZr hydrogel fibers were able to dissipate more energy with increasing dissipation efficiency.

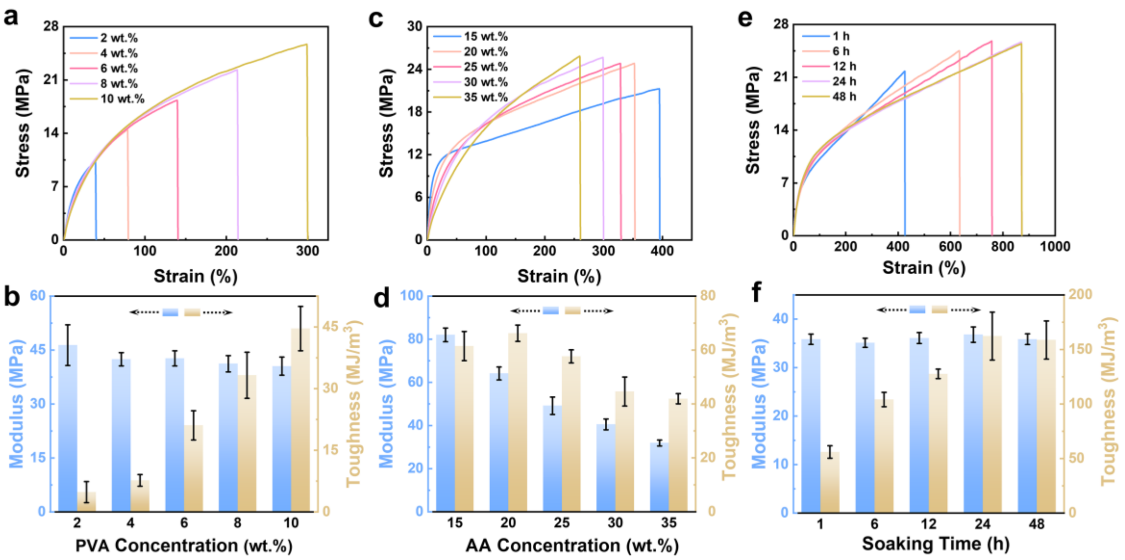

Supplementary Figure 6. Effect of (a-b) PVA concentration, (c-d) AA concentration, and (e-f) salt-out treatment time on the mechanical properties of S-PAZr hydrogel fibers. S-PAZr hydrogel fibers were toughened with increasing PVA concentration due to more possible formation of crystal domains. As increasing AA concentration, the ratio of AA to Zr^{4+} raised and interfered more with PVA crystallization, resulting in lower elastic modulus and tensile strain of S-PAZr hydrogel fibers. The mechanical properties of S-PAZr hydrogel fibers were gradually toughened and stabilized with increasing soaking time. The insignificant change in elastic modulus of S-PAZr hydrogel fiber with increasing soaking time may be attributed to the fact that the unstable crosslinked Zr^{4+} has been displaced from the hydrogel fiber into the ternary solvent within 1 h. Data were presented as mean ± SD (n = 3 independent samples). Notes: when exploring the effect of PVA concentration on the mechanical properties of S-PAZr hydrogel fibers, the AA concentration was 30 wt.%, the Zr^{4+} concentration was 0.4 M, the Na_2SO_4 concentration in the ternary solvent was 2 M, and the soaking time was 24 h. When exploring the effect of AA concentration on the mechanical properties of S-PAZr hydrogel fibers, the PVA concentration was 10 wt.%, the Zr^{4+} concentration was 0.4 M, the Na_2SO_4 concentration in the ternary solvent was 2 M, and the soaking time was 24 h. When exploring the effect of soaking time on the mechanical properties of S-PAZr hydrogel fibers, the PVA concentration was 10 wt.%, the AA concentration was 20 wt.%, the Zr^{4+} concentration was 0.3 M, and the Na_2SO_4 concentration in the ternary solvent was saturated.

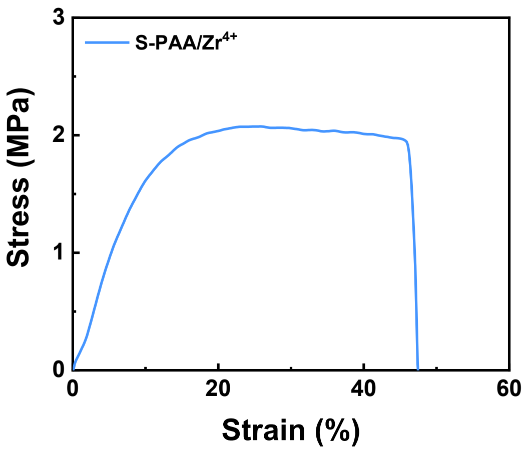

Supplementary Figure 7. Mechanical properties of S-PAA/Zr^{4+} hydrogel fibers without Hofmeister effect-sensitive polymers. S-PAA/Zr^{4+} hydrogel fibers exhibited tensile stress of 1.93 ± 0.29 MPa and tensile strain of 43.09 ± 10.17%. Data were presented as mean ± SD (n = 3 independent samples)

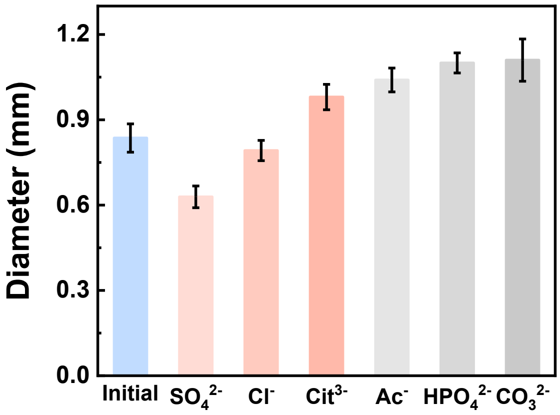

Supplementary Figure 8. Diameters of PAZr hydrogel after immersion in Gly/H_2O solutions of 1 M Na_2SO_4, NaCl, Na_3Cit, NaAc, Na_2HPO_4, and Na_2CO_3 for 24 h. PAZr hydrogel fibers immersed in a ternary solvent containing Na_2SO_4 and NaCl underwent shrinkage due to additional crystalline domain crosslinking and H_2O outfflux caused by the highly concentrated salt solution. Owing to the alkaline environment caused by the ternary solvent containing Na_3Cit, NaAc, Na_2HPO_4, and Na_2CO_3, the ionic coordination in the hydrogel fibers was uncoordinated leading to swelling of hydrogel fibers. Data were presented as mean ± SD (n = 3 independent samples).

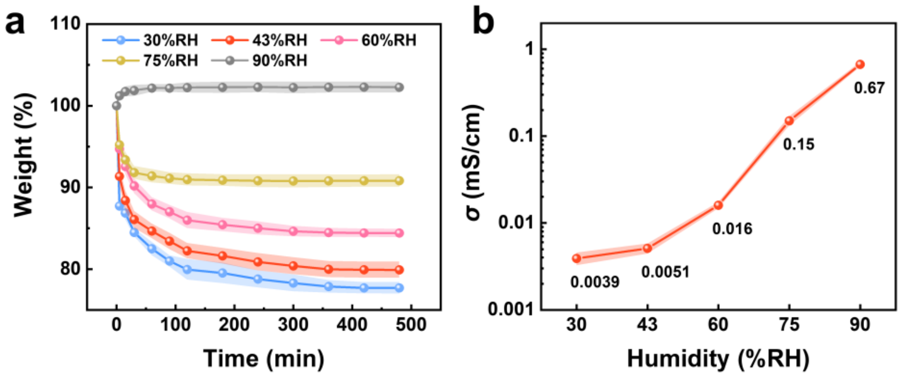

Supplementary Figure 9. (a) The weight and (b) conductivity of S-PAZr hydrogel fibers at different humidity. The equilibrium mass and conductivity of S-PAZr hydrogel fibers were improved with increasing humidity. Data were presented as mean ± SD (n = 3 independent samples).
